# Observations on the Ætiology and Pathology of Odontatrophia

**Published:** 1851-01

**Authors:** Chapin A. Harris


### ARTICLE III.

### *Observations on the Ætiology and Pathology of Odontatrophia.*

By CHAPIN A. HARRIS, M. D.

To the peculiar affection of the teeth on which we at this time propose offering a few remarks, M. Duval, a French dentist, and author of some celebrity, has applied the term atrophy. But the strict applicability of the term, as the two principal varieties of the affection consist rather in a congenital defect, most frequently, of some portion of the enamel of two or more of the teeth, than wasting, from want of nourishment, of any of the dental tissues, may, perhaps, be considered as somewhat questionable. And this would seem to be rendered still more so by the fact, that neither of the varieties to which we have referred, occurs subsequently to the formation of the enamel. But as the congenital form of the disease is evidently the result of altered function in a portion of one or more of the formative organs, if not of absolute degeneration, from vicious nutrition, we are disposed to regard the term as the most applicable of any that can be applied to it.

The occurrence of odontatrophia is less frequent than any other disease to which the teeth are liable, and as the progress of the affection usually ceases with the termination of the causes concerned in its production, it has scarcely been deemed of sufficient importance to merit serious consideration. Hence, neither its ætiology nor pathology has been very carefully investigated. Indeed, most writers upon the diseases of the teeth have overlooked the affection altogether, while a few have only merely alluded to it, without describing the characteristics even of its principal varieties, and although we have treated of it in another place, the limits to which we there felt ourself restricted, did not admit of a very full discussion of the subject.* Whether we shall now be able to throw any additional light upon it, or establish the correctness of any opinions already advanced, or not, we shall leave to others to determine.

In the formation of the enamel, Mr. Fox speaks of a defect, sometimes met with in its organization, characterized by a "yellow color," and of "a great number of indentations upon its surface," giving to the teeth the appearance of "the exterior of a sponge," which he termed "honey-combed." He refers these defects to a deviation from the natural action of the membrane which secretes the enamel, and dependent upon some "peculiarity of constitution," during the first months of infancy. He thinks it is liable to occur in several children of the same family, and that teeth thus affected are less liable to decay than those which have beautiful and transparent enamel.†

M. Delabarre, an ingenious physiologist, and for the most part, a close and very accurate observer, has, probably, approached nearer a correct explanation of the true cause of dental atrophy than any other writer. But he has evidently confounded one of the varieties of the affection with erosion—a disease characterized by essentially different phenomena, and produced by different causes. He restricts the term to the variety which consists simply of discoloration and absence of a portion of the enamel. In the former cases he believes it may be congenital or accidental, but in the latter, he thinks it is always congenital. He confounds the variety which consists of perforations in the enamel with erosion.‡ But, so distinctly

* Vide Principles and Practice of Dental Surgery, p. 337, 4th ed.

† Natural History and Diseases of the Human Teeth; American edition, pages, 57, 8 and 9.

‡ Traitè de la Seconde Dentition, pp. 20, 1, 2 and 3.

marked are the peculiarities of each, there is no necessity for confounding the one with the other.

The opinion of M. Delabarre with regard to the cause of dental atrophy, is founded upon the supposition, that the doctrine of the formation of the enamel as maintained by Hunter, Jourdain, Fox and Cuvier, is erroneous, and that this portion of a tooth is formed from an immense number of exhalents which cover the crown, forming a "sort of imperceptible velvet."* These he regards as integral parts of the tooth, and believing the enamel fibres to be secreted by them, he ascribes the affection under consideration to their vicious development or imperfect nutrition.

Lefoulon adopts the views and almost the precise language of Delabarre, in the description which he gives of atrophy of the teeth. Maury treats of both it and erosion as being one and the same disease. But in describing atrophy he notices the distinctive peculiarities by which each affection is characterized.†

In describing the difference between erosion and atrophy, M. Delabarre says, the part atrophied is deformed and deprived of the enamel, "that the teeth are yellow and sensitive, the touch of the finger causing pain." In erosion, if the crystals of the enamel are not wholly destroyed, "the bottom of it is of a white color, and on its being touched, causes no disagreeable sensation; if, on the contrary, the crystals are destroyed down to the tooth, the part thus denuded is irritable."

In an article on erosion, Maury gives a very accurate description of several varieties of atrophy of the teeth. The first, he represents as consisting of irregular white, deep or light yellow spots, situated in the enamel of the tooth, without affecting the smoothness of its surface. The second; as characterized by little crowded holes, or irregular depressions, resemb-

* It is scarcely necessary to state that the exhalents of Delabarre, correspond exactly with the corpuscules or fibres of the enamel membrane of Raschkow.

† Traitè Complet de l'Art du Dentiste, pp. 99 and 100.

ling quilting; or as consisting of transverse sinuosities, single or divided by prominent lines, which are sometimes "yellow, but of the color of the enamel." The third variety affects the dentine as well as the enamel, reducing the dimensions of the crown of the tooth sometimes to one third of its natural size, and not unfrequently dividing it by a deep circular groove or depression.

Not one of the phenomena here described is produced by the action of corrosive agents, or is the result of chemical decomposition either of the enamel or dentine, but are manifestly dependent upon other causes. The term erosion, therefore, cannot be applied to either variety of the affection just noticed, with the least show of propriety.

Odontatrophia may very properly be divided into three varieties. Each has distinctive peculiarities which characterizes it from either of the others. Two are always congenital, and the other, although most frequently congenital, sometimes occurs subsequently to the eruption of the tooth.

Although Maury has given a better description of the three principal varieties of dental atrophy than any other writer, he has omitted some things which it will be proper to mention. But in treating of these different varieties, we shall change, somewhat, the order of the arrangement which he has laid down.

*First variety.* The peculiarities that distinguish this variety of atrophy from either of the others are, that it never impairs the uniformity and smoothness of the surface of the enamel, and is characterized by one or more white, or dark or light brown, irregularly shaped spots, upon the labial or buccal surface of the tooth. It occurs oftener than the third variety, and less frequently than the second. It rarely appears on more than one or two teeth in the same mouth, though several are sometimes marked by it. It is seen on the molars more frequently than the bicuspids, and much oftener on the incisors of the upper jaw than any of the other teeth. We do not recollect to have ever observed it on the cuspids of either jaw, nor on the palatine or lingual surfaces of the incisors.

The enamel is much softer on the affected than on the unaffected parts of the tooth, and may be easily broken and reduced to powder with an instrument. It seems to be almost wholly deprived, in these places, of its animal constituents as well as its connection with the subjacent dentine.

The size of the atrophied spots are almost as variable as their shape, but the only bad effect produced by them, is the unpleasant aspect they sometimes give to the tooth.

As we have before remarked, this variety of atrophy is sometimes accidental, occurring subsequently to the eruption of the tooth, but in a large majority of the cases it is congenital. It is rarely seen on a temporary tooth. In all the cases which have come under our observation it was confined to the teeth of second dentition.

*Second variety.* This may be very properly denominated *perforating* or *pitting* atrophy, as it gives to the enamel an indented or pitted appearance, the irregular depressions or holes extend transversely across and around the tooth. They are sometimes more or less distinctly separated one from another, by prominent lines; at other times they are confluent, and form an irregular horizontal groove. Sometimes they penetrate but a short distance into the enamel; at other times they extend entirely through it to the dentine. Their surface, though generally rough and irregular, usually presents a glossy and polished appearance—a peculiarity which always distinguishes this variety of the affection from erosion. The pits often have a dark brownish appearance, though sometimes they are of the color of the enamel on other parts of the tooth.

This variety of atrophy is never confined to a single tooth, Two, four, six or more corresponding teeth are always affected at the same time, in each jaw, and the corresponding teeth of the same class precisely in the same manner, and in the same place. When more than two are marked, the distance of the pits from the coronal extremities of the organs varies, according to the progress the formation of the enamel had made on each class at the time of the operation of the causes concerned in the production of the affection. For example, when the line of pits in the central incisors is situated about two lines from their cutting edges, it will scarcely be one line from the cutting edges of the laterals, and only the points of the cuspids will be marked. When the indentations are nearer the edges of the central incisors, they will be on the edges of the laterals, and the cuspids will have entirely escaped.

Sometimes the teeth are marked with two or three rows of pits, and when this is the case, the patient has either had two or three relapses or been attacked two or three times in quick succession with different diseases capable of interrupting the progress of the formation of the enamel.

Although the incisors are more frequently marked with these indentations than any of the other teeth, the cuspids, bicuspids, and even the molars are sometimes affected with them. When the disease attacks the molars, its effects are generally located on the grinding surface. The permanent teeth are more liable to be attacked than the temporary. We have never known but one instance in which the latter were affected with the disease.

This variety of atrophy occurs oftener than either of the others, and though it sometimes gives to the teeth a very disagreeable and unsightly appearance, it rarely increases their liability to decay.

*Third variety.* In this variety of atrophy the whole or only a part of the crown of a tooth may be affected—the dentine being often implicated as well as the enamel. The tooth usually has a pale yellowish color, a shrivelled appearance, and is partially or wholly divested of the enamel. Sometimes the crown is not more than one-half or one third its natural size. Its sensibility is usually very greatly increased, and its susceptibility to pain from external impressions is most wonderfully excited by acids. It is also more liable than the other teeth to be attacked by caries. The root of the tooth, though rarely, is nevertheless sometimes affected, and presents an irregular knotted appearance.

The disease is often confined to a single tooth, but it more frequently shows itself on two corresponding teeth in the same jaw. According to our observation, the bicuspids are more liable to be attacked than any of the other teeth. The temporary teeth are rarely affected with it.

This variety of atrophy occurs less frequently than either of the others, and although it increases the liability of the affected organs to decay, they sometimes escape its attacks to an advanced period of life.

In the description which we have given of the three varieties of dental atrophy, we may have omitted to mention some of the peculiarities belonging to each, but we believe we have pointed out their principal characteristics with sufficient accuracy to enable almost any one to distinguish one from another, and either from erosion. We shall, therefore, conclude our remarks upon this subject with a few observations on the ætiology of each variety of the disease.

The first variety is evidently produced by some cause capable either of preventing or destroying the bond of union between the enamel and subjacent dentine, but what that cause always is, is a question which it may be difficult to answer. Subsequently to the eruption of the teeth, it may be occasioned by mechanical violence, but we have never known but one case in which it had resulted from this cause. The subject of this was a boy about ten or eleven years of age; he accidentally fell while running, and struck the right central incisor against a sharp angle of a rock. The tooth was nearly displaced, and remained sore and sensitive to the touch for several weeks. Ultimately a small white spot developed itself on the labial surface of the organ.

Now, whether the bond of union between this portion of the enamel and the subjacent dentine was immediately destroyed by the concussion of the blow, or whether it resulted from subsequent inflammation and the death of the intermediary membrane, is a question we do not feel ourself able to answer. If it were destroyed at once by the blow, one would be led to suppose that the change in the color of the enamel would have been observed immediately, but this may have resulted from some subsequent change or alteration in the animal constituents of this part of the enamel, which followed as a consequence of the injury produced by the violence of the blow. These are questions, however, which the present state of our knowledge does not enable us to solve. But that the white spot in this case resulted as a consequence of the blow, there cannot be the least shadow ofdoubt.

But when the affection is congenital, as it almost always is, it is dependent upon some other cause, possibly upon disease in the pulp, or intermediary membrane, which constitutes the bond of union between the dentine and enamel, subsequently to the formation of the latter. But what the determining cause of the disease in these parts, is, if the affection be produced in this way, whether simple local irritation, or general constitutional disturbance, we are not prepared to say. One would be likely to suppose, if the atrophied spots were occasioned by disease of the pulp or intermediary membrane, the morbid action would scarcely confine itself to such narrow and circumscribed limits. But, whether the destruction of the intermediary membrane of the affected part results as a consequence of actual disease, or merely from vicious nutrition, or whether from some unknown cause, it has failed to be developed here, it is certain that the fibres of this portion of the enamel are not united to the subjacent dentine; and, not receiving a supply of nutrient fluid or vital principle, their animal frame-work partially or wholly perishes, leaving but little more than their inorganic constituents.

The cause of this variety of congenital atrophy, it must be confessed, is very obscure; and in the absence of positive knowledge, we can only infer it from the nature of the affection. If it does not result from one or other of the above mentioned causes, it is difficult to imagine in what way it is produced.

The cause of the second variety of odontatrophia is, we think, susceptible of a more satisfactory explanation. The formative organ of the enamel, as is now generally admitted, consists of a membrane, composed almost wholly of short hexangular corpuscules or fibres, which correspond in shape and arrangement with the fibres of the enamel. This membrane is accurately moulded to the crown of the tooth, and according to

Raschkow, each fibre is a secretory duct, whose peculiar function it is to secrete the fibre of the enamel corresponding to it. It should also be borne in mind that the secretion of the earthy salts of the enamel commences at the coronal extremity of the tooth, and gradually proceeds towards the base of the crown. Now we can readily conceive that some constitutional disease, by interrupting the secretion of the earthy salts deposited in the enamel cells or secretory ducts of the enamel membrane, for the formation of the enamel fibres, by occurring at the time when this process is going on, might prevent them from being filled, and cause them to wither or waste away, giving to this portion of the enamel the pitted appearance which characterizes this variety of atrophy. In other words, the secretion of the inorganic constituents of the enamel being interrupted for a short time, the horizontal row of cells in the enamel membrane, into which it should be deposited, will not be filled, consequently, as we might readily suppose, they will waste away, leaving a circular row of indentations around the crown of the tooth. But as soon as the constitutional disease has run its course, the secretion of the enamel fibres will be resumed, and unless the child experiences a relapse, or has a second attack of disease capable of interrupting this secretory process, the other parts of the enamel will be well formed.

Some writers ascribe the formation of these pits in the enamel to the chemical action of a corrosive fluid, or to an acidulated condition of the fluid contained in the dental sacs, but those who have done so, have confounded the affection with erosion. Others, and with much plausibility, believe they result as a consequence of an eruptive disease during the "enameling" process, and there are many facts which go to sustain the correctness of this opinion. In seventy-one cases which we have recorded, that have come under our observation during the last eighteen years, we have endeavored to elicit all the facts which we supposed could have any connection with the origin and cause of the disease. The following is the result of our inquiries.

In thirty-eight cases, we were informed, either by the individuals themselves, or relatives, that they had had the measles, and as nearly as we could ascertain, sometime during the formation of the enamel of the teeth : in six cases, the chicken pox; in four, scarlet fever; in three catarrhal fever; in two small pox; in one the mother had had an attack of varioloid about seven or eight weeks previous to her accouchment, but in this last case, the atrophied teeth belonged to the temporary set. With regard to the other seventeen cases, we were unable to obtain any certain reliable information concerning the state of the general health during the few first years of childhood.

Although the information we obtained concerning the early history of the fifty-four cases, was not, in many instances, very precise in relation to the exact age when the diseases we have mentioned occurred, it was sufficiently so, we think, to establish their probable connection with the condition of the teeth, or rather, their agency in the production of the atrophy.

Three cases occurred in the same family in two instances, and two cases in six. How far this fact may go to establish the correctness of the opinion advanced by Mr. Fox, that several cases are liable to occur in the same family, we leave to others to decide. If eruptive diseases are more favorable to the production of this variety of atrophy than others, the correctness of it might seem to be favored by the consideration, that when one of this class of diseases occurs in a family, several of the children, where there are a number, are usually attacked about the same time; and when it happens that there are two of nearly the same age, the teeth of both would be likely to be affected, those of the youngest nearer the coronal extremity than the other. But the differences in the ages of children in the same family, would, in the majority of cases, prevent the teeth of more than one from being marked with atrophy, unless eruptive diseases should occur at different times in the family.

The cases to which we have referred, would seem to establish the fact, that although the affection is caused altogether, and by far more frequently, by eruptive, than any other form of constitutional disease, it may, nevertheless, occasionally result from other diseases.

The third variety of dental atrophy, so far as our observations upon the subject would seem to justify an opinion, always results from altered or vicious nutrition, caused by diseases of the pulp or enamel membrane, or both, during the secretion of the dentine or enamel, according as one or both are affected. We are inclined to believe that the disease in the dental pulp or enamel membrane may be produced either by local or constitutional causes, or both. But the information which we have been able to obtain concerning the state of the general health, and that of the mouth at the time of the ossification of the pulp and the secretion of the enamel, in the cases that have fallen under our observation, has not been as satisfactory as we could have wished.

In only thirteen out of nineteen cases, of which we have kept a record, were we able to elicit any information calculated to throw the least light upon the origin and cause of the affection. In five of these cases, we ascertained, upon inquiry, that the gums around the temporary teeth had been very much diseased from alveolar abscess, for about three years and a half or four years previously to the eruption of the organs of replacement, and that purulent matter had been discharged from them a great portion of this time. The subjects of three of the other cases had a severe attack of dysentery about the third year of age, and from the effects of which they did not recover for a long time. In two cases, the subjects had been badly salivated, and in consequence of which, lost two of the temporary teeth, from necrosis and exfoliation of their sockets, about the third year of age. One of these last was almost wholly deprived of enamel, of a light brown or yellow color, and but little more than half the natural size. The subjects of two of the other cases had had a severe attack of fever, about the time, as nearly as we could ascertain, of the commencement of the ossification of the atrophied teeth, and from the effects of which they did not recover for eight or ten months. The subject of the other case had had several attacks of sickness between the first and seventh year of age, which so impaired the general health, as to render its restoration a matter of great doubt, both with the parents and attending physician.

In four out of the nineteen cases, the crowns of the teeth were not much more than one-half the natural size, with scarcely a single trace of enamel on them, and three were of a light, and one of a dark brown color. They were exceedingly sensitive to the touch, and three were badly decayed in their approximal surfaces, rendering their immediate removal absolutely necessary. The portion of the dentine of these which was not affected by caries, was so soft that it could be easily cut with a knife. Their roots were feebly developed, and in other respects more or less imperfect.

The crowns of the teeth, in five of the other cases, had a shrivelled appearance, were much smaller than the natural size, with only small patches of enamel on the cusps and points of their coronal extremities, which were the only parts not preternaturally sensitive to the touch. The other parts were of a pale or dark brown color. Four of them were affected with caries in their approximal surfaces, and in two it had extended to the pulp-cavity, rendering their removal necessary.

From one-fourth to one-half of the crown, in the other ten cases, was either wholly deprived of enamel, or covered with only a very thin discolored layer, and of so soft a texture that it could have been easily scraped away with a knife or other instrument. The atrophied parts of six were affected with caries.

Eleven out of the nineteen teeth were bicuspids—eight in the lower and three in the upper jaw; one was a superior cuspidatus; three were incisors—two in the upper and one in the lower jaw; and four were molars. In eighteen of the cases, the atrophy was confined to the permanent teeth.

The circumstances which we have mentioned, as having had a probable connection with the origin and cause of the atrophy, in the thirteen cases to which we have particularly referred, would seem to warrant the conclusion, that altered function or vicious nutrition of the pulp or enamel membrane, or both, upon which the structural peculiarities or morbid phenomena presented by teeth thus affected, are dependent, is the result of either local or constitutional disease. Although we failed, in six cases, to elicit any information concerning either the state of the general health, or the condition of the mouth, when the secretion of the earthy salts of the atrophied teeth must have been going on, it is certain that this process must have been interfered with, either by some local or constitutional disturbance.

The difficulty of obtaining a correct account of all the circumstances connected with the history of any disease, several years after its occurrence, must, when the inquiry is delayed for so long a period, prevent us from becoming sufficiently acquainted with them to determine the exact importance to which each is entitled. This has ever constituted a serious obstacle to the investigation of obscure causes of morbid phenomena; but in the examination of the affection on which we have been treating, we believe the facts we have adduced justify the inferences we have ventured to make.

Since writing the foregoing, the following interesting case of dental atrophy has fallen under our observation:

Mrs. C. called, December 16th, 1850, to consult us concerning her daughter's teeth, which, from congenital defect, presented a most unsightly appearance. The girl was between nine and ten years of age. The cutting edges of the upper central incisors were badly pitted and very rough; the corresponding teeth in the lower jaw had a transverse row of pits passing around them, about a sixteenth of an inch below their cutting extremities. Another row of pits, so close together as to form a rough groove, encircled the upper central incisors, about an eighth of an inch below the gum, and the laterals a little nearer their cutting edges; the lower incisors were similarly marked, but not quite so near the gum. The enamel, near the second transverse row of pits, and between it and the incisive extremities of the teeth, was thin, and of a light brown color. A little above the first row, on the central incisors, were two or three brown, opaque spots. The first permanent molars were also encircled with a row of indentations, about half way between their grinding surfaces and the gums.

On inquiry, we learned from the mother, that the child had a light attack of measles when she was between eleven and twelve months old; of scarlet fever when about fifteen or sixteen months of age, and dysentery at about the twenty-first or twenty-second month.

Now, here we have the three varieties of atrophy on the same teeth, and the occurrence of constitutional disease about the time when the affected parts of the teeth must have been receiving their earthy salts, would seem to establish, very conclusively, the connection of the one with the other.

